# Dental-Derived Stem Cells and Their Secretome and Interactions with Bioscaffolds/Biomaterials in Regenerative Medicine: From the In Vitro Research to Translational Applications

**DOI:** 10.1155/2017/6975251

**Published:** 2017-12-28

**Authors:** Andrea Ballini, Antonio Boccaccio, Rajiv Saini, Phuc Van Pham, Marco Tatullo

**Affiliations:** ^1^Department of Basic Medical Sciences, Neurosciences and Sense Organs, University of Bari Aldo Moro, Bari, Italy; ^2^Department of Mechanics, Mathematics and Management, Politecnico di Bari, Bari, Italy; ^3^Department of Periodontology and Oral Implantology, Pravara Institute of Medical Sciences, Loni, Maharashtra, India; ^4^Laboratory of Stem Cell Research and Application, University of Science, Vietnam National University, Ho Chi Minh City, Vietnam; ^5^Biomedical Section, Tecnologica Research Institute, Crotone, Italy

Regenerative dentistry is an innovative field of medicine that is growing involving both dental and maxillofacial sciences [1, 2].

Clinical healing occurs when new regenerated tissue is well integrated into the previously damaged host tissue: in this context, the reparative and regenerative actions of resident and recruited mesenchymal stem cells (MSCs) have been thoroughly performed.

In the most recent literature, the MSC-produced secretome has been widely studied and it has been even more considered as the strategic promoter of the vast majority of the biological effects derived from stem cell transplantation [3–5].

Dental-derived mesenchymal stem cells (D-dMSCs) are today considered as an intriguing milestone of the regenerative medicine as such cells have been reported to have a strong ability to differentiate into osteogenic, adipogenic, and chondrogenic lineages, with a peculiar ability to improve the bone mineralization [6–9].

Complete healing might be achieved by establishing novel strategies, by using scaffolds in combination with oral-derived MSCs in the presence of secretome and growth [3].

The interaction between stem cells and biomaterials is a crucial topic; recent research trends were focused and developed on the interaction both at superficial macroscopic level and at structural microscopic level. About the first ones, involving the researches on scaffold-related macroscopic features, there are evidences that geometrical and mechanical properties of scaffolds are able to influence the cell behavior and their response to differentiating stimulations [10].

Among the manufacturing processes that can be used to fabricate biomimetic scaffolds, the strategy based on the combination of additive manufacturing and computer-aided design (CAD) modelling seems to be one of the most promising [11]. The possibility to design and create any shape for the newly produced scaffolds, and the scientifically confirmed evidence that scaffold geometry plays a crucial role in influencing the MSC response, led researchers towards an increasing attention to scaffold design; more in details, bioengineers designed complex morphologies able to be reproduced on the surfaces of porous biomaterials [12–15].

Other types of research studies were related to microscopic features of scaffolds, demonstrating that many changes in scaffold microarchitecture modified, for example, the adhesion of stem cells to the scaffold surfaces [16]. The adhesion of stem cells to scaffold is a biologically guided result of complex cellular, physical, and chemical processes, and it is an essential requirement to guarantee a proper and effective tissue engineering aimed to healing and regenerative applications. Differently from the huge number of studies focused on biochemical reactions that trigger stem cell differentiation, very few studies are reported in the scientific literature about how the mechanical environment affects the adhesion of stem cells on biomaterials' surfaces [17, 18].

We believe that extensive studies will be carried out on this topic in the next few years. However, much still needs to be elucidated in order to be able to create efficient and safe bioartificial substitutes for clinical use.

This special issue has reported articles on D-dMSCs used as therapeutic aid in clinical and surgical applications. The human dental pulp stem cells (hDPSCs) seem to be still the most used cell model by the SI authors.

The most reported translational use of D-dMSC therapy is related to tissue regeneration: in fact, authors have investigated about cytotoxicity, genotoxicity, and biocompatibility of endodontic materials for hDPSCs (A. Victoria-Escandell et al.) or compared using this cell line the efficiencies of osteogenic differentiation and *in vivo* bone formation of hydroxyapatite-tricalcium phosphates (HA-TCPs) and demineralized dentin matrix (DDM) (K.-J. Kang et al.).

An interesting general view has been also given on topics related to issues of general interest, as the potential effect of heavy ethanol consumption can inhibit odontogenic differentiation, a factor that needs to be considered in clinical practice during pulp therapy (W. Qin et al.).

Moreover, other authors have focused their researches to consider that nuclear receptor related 1 (NURR1) plays a key role in switching hDPSC differentiation towards osteoblast rather than neuronal or even other cell lines (A. Di Benedetto et al.).

Finally, some authors have also reported interesting aspects about the role of nephronectin (Npnt) to recruit and conducive to mineralization in hDPSCs, offering a promising approach for hard tissue regeneration (J. Tang and T. Saito).

In this special issue, the editors together with the involved authors have well described the D-dMSCs in their different but fundamental roles of promoters, enhancers, and playmakers of the translational regenerative medicine.

Starting from the contents of our issue, the scientific community will be stimulated to experiment new ideas, to improve the knowledge of D-dMSCs, and to speed up their clinical application, so to improve regenerative medicine approaches.

*Andrea Ballini* *Antonio Boccaccio* *Rajiv Saini* *Phuc Van Pham* *Marco Tatullo*
